# Supplementary material for: Function-preserving CD8+ T Cell Immunogenicity minimization through protein design
Source: bioRxiv. 2026 Oct 27:2026.10.24.761497. Preprint. [Version 1] doi: 10.64898/2026.10.24.761497 (PMC13733841; doi:10.64898/2026.10.24.761497)
Supplement: Supplementary file 1 [file NIHPP2026.10.24.761497V1-supplement-1.pdf]

bioRxiv preprint doi: https://doi.org/10.64898/2026.10.24.761497; this version posted October 27, 2026. The copyright holder for this preprint (which was not certified by peer review) is the author/funder, who has granted bioRxiv a license to display the preprint in perpetuity. It is made available under a CC-BY 4.0 International license.

Figure S1. Sequence diversity and predicted MHC-I epitope burden of LuxSit-i variants

(A) Sequence entropy of each site illustrates amino acid diversity at each position among LuxSit-i sequences generated from MINT with no cycling. Red color indicating the fixed residues.

(B) Distribution of the number of strong (EL rank <0.5%) MHC-I epitopes for each of 5 mouse MHC-Is (left) and each of 25 human MHC-Is (right) among LuxSit-i sequences generated from MINT with no cycling.

Figure 2. Designed luciferase variants elicit distinct CD8$^+$ T cell responses in vivo

(A) Schematic of the 5+1 fusion constructs containing five predicted low-immunogenicity variants and one predicted immunogenic LuxSit-i variant.

(B) Schematic of the mRNA-LNP immunization regimen and subsequent assessment of antigen-specific CD8$^+$ T cell responses.

bioRxiv preprint doi: https://doi.org/10.64898/2026.10.24.761497; this version posted October 27, 2026. The copyright holder for this preprint (which was not certified by peer review) is the author/funder, who has granted bioRxiv a license to display the preprint in perpetuity. It is made available under a CC-BY 4.0 International license.

(C) Frequencies of IFN-γ$^+$ CD8$^+$ T cells responding to individual LuxSit-i variants following immunization with 5+1 fusion constructs.

(D) Top: schematic of the individual-variant immunization experiment. Bottom: frequencies of IFN-γ$^+$ CD8$^+$ T cells responding to individual LuxSit-i variants following immunization.

(E) Cross-reactivity of CD8$^+$ T cell responses elicited by individual immunogenic LuxSit-i variants against other immunogenic variants.

Figure S2. Expression and characterization of CD8$^+$ T cell responses to designed luciferase variants

(A) mScarlet reporter expression in bone marrow-derived dendritic cells (BMDCs) transfected with mRNAs encoding individual LuxSit-i variants.

(B) Representative flow cytometry plots showing IFN-γ production by CD8$^+$ T cells following immunization with 5+1 fusion constructs.

(C) Frequencies of TNF-α$^+$ CD8$^+$ T cells responding to individual LuxSit-i variants following 5+1 fusion immunization.

(D) Frequencies of IL-2$^+$ CD8$^+$ T cells responding to individual LuxSit-i variants following 5+1 fusion immunization.

(E) Thy1.1 reporter expression in bone marrow-derived dendritic cells (BMDCs) transfected with mRNAs encoding individual LuxSit-i variants.

Table S1. Sequences and numbers of predicted immunogenic peptides for the 10 LuxSit-i variants used in the in vivo experiments.

bioRxiv preprint doi: https://doi.org/10.64898/2026.10.24.761497; this version posted October 27, 2026. The copyright holder for this preprint (which was not certified by peer review) is the author/funder, who has granted bioRxiv a license to display the preprint in perpetuity. It is made available under a CC-BY 4.0 International license.

Figure S1

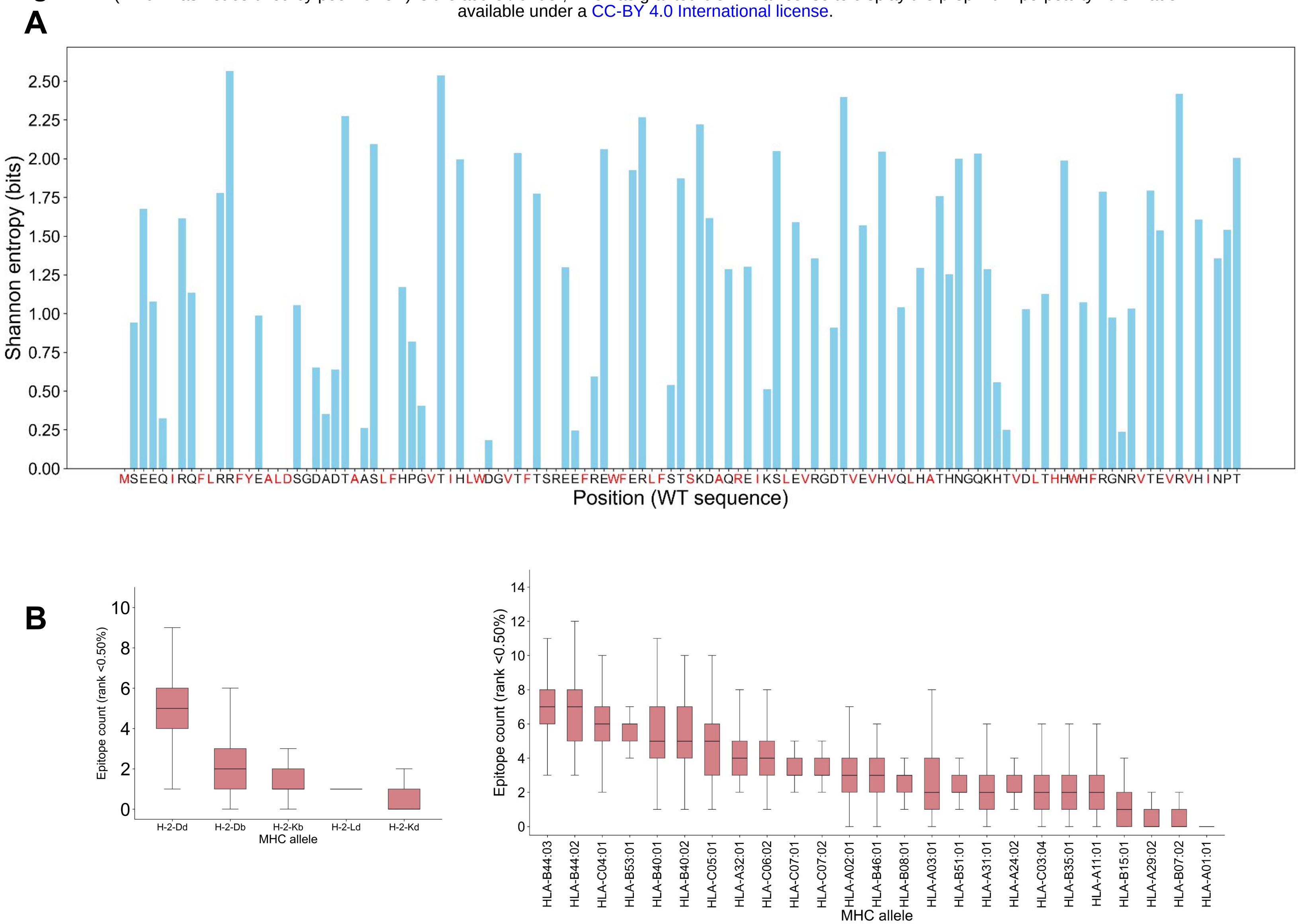

bioRxiv preprint doi: https://doi.org/10.64898/2026.10.24.761497; this version posted October 27, 2026. The copyright holder for this preprint (which was not certified by peer review) is the author/funder, who has granted bioRxiv a license to display the preprint in perpetuity. It is made available under a CC-BY 4.0 International license.

Figure S2

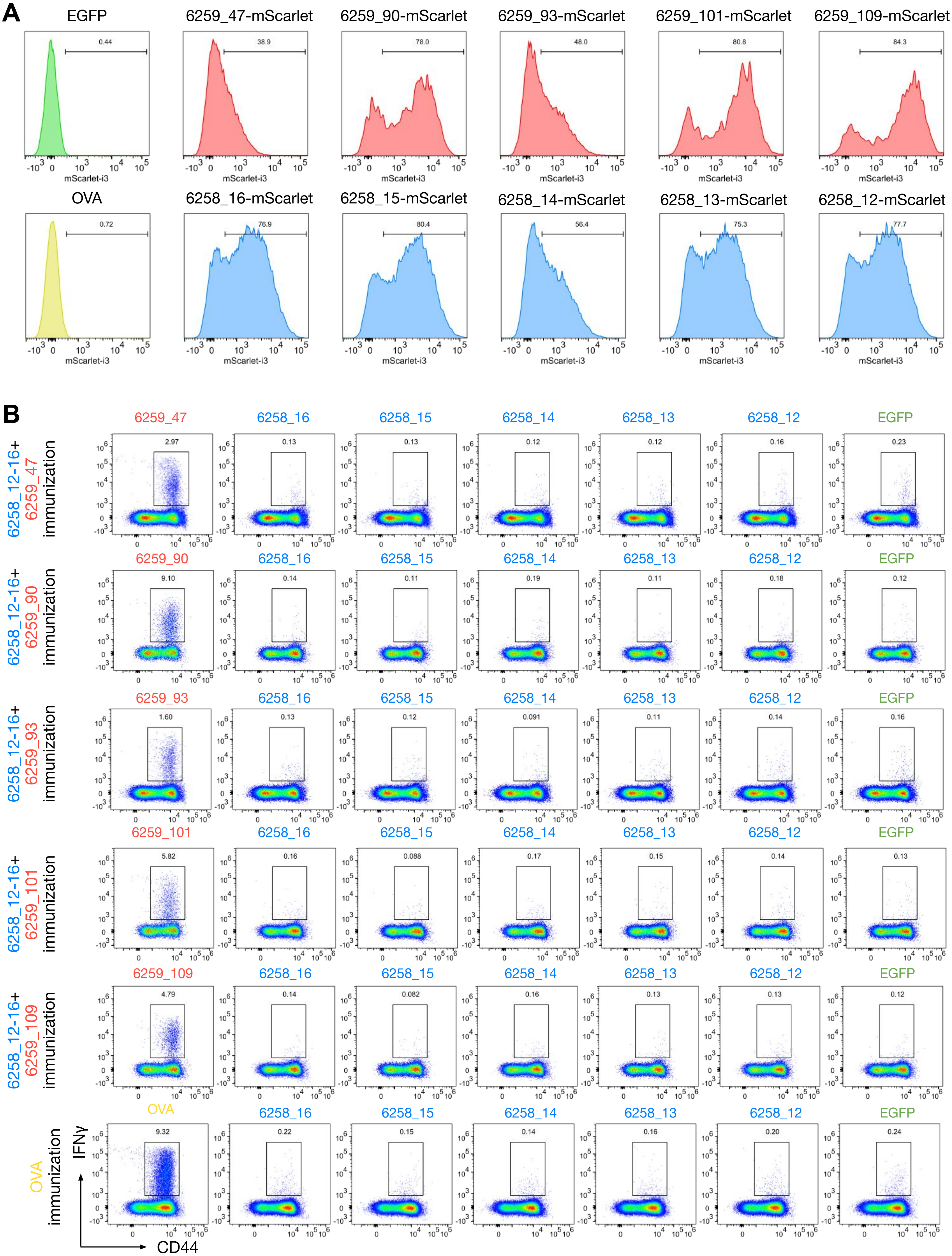

bioRxiv preprint doi: https://doi.org/10.64898/2026.10.24.761497; this version posted October 27, 2026. The copyright holder for this preprint (which was not certified by peer review) is the author/funder, who has granted bioRxiv a license to display the preprint in perpetuity. It is made available under a CC-BY 4.0 International license.

Figure S2

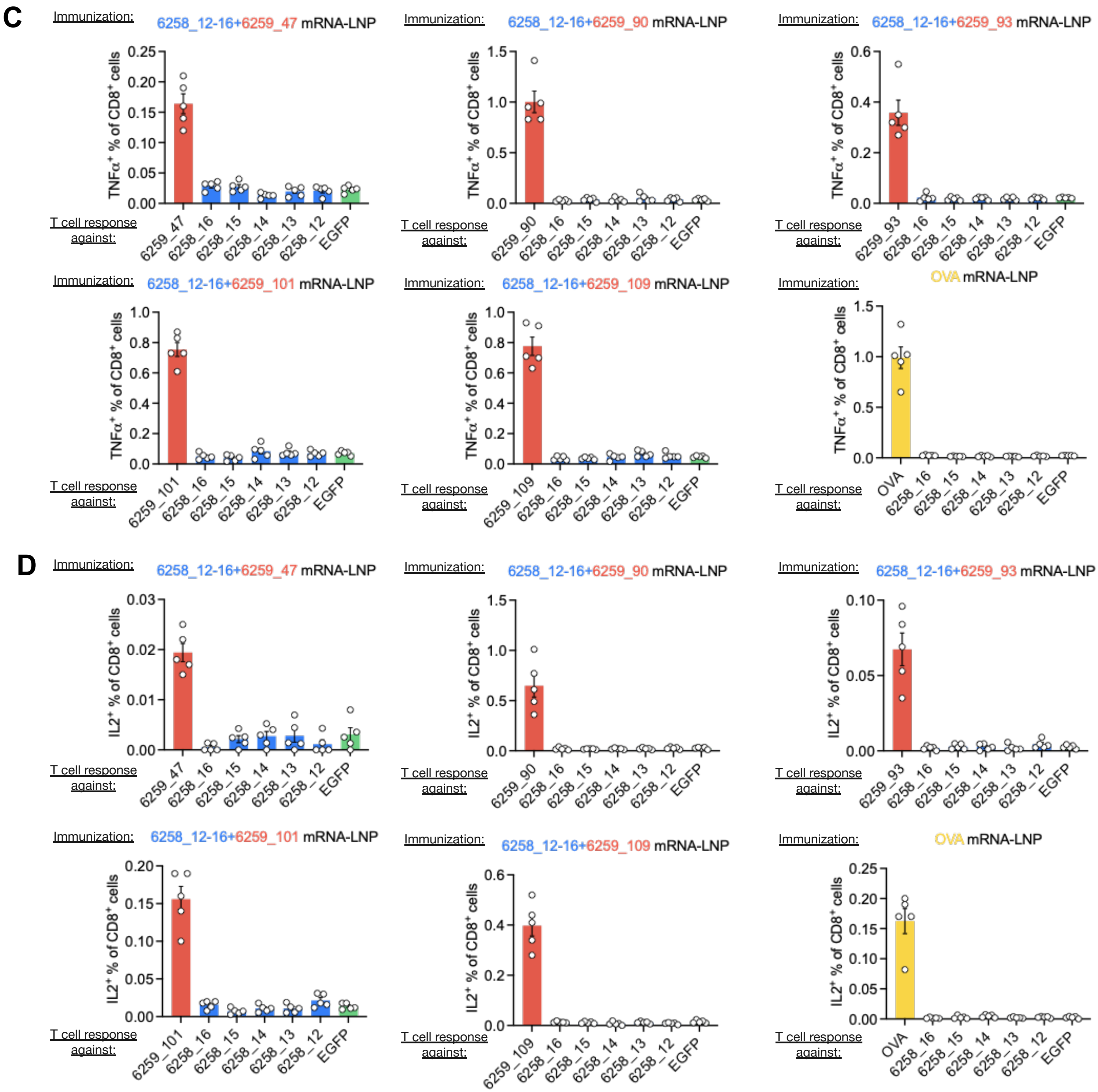

Figure S2

bioRxiv preprint doi: https://doi.org/10.64898/2026.10.24.761497; this version posted October 27, 2026. The copyright holder for this preprint (which was not certified by peer review) is the author/funder, who has granted bioRxiv a license to display the preprint in perpetuity. It is made available under a CC-BY 4.0 International license.

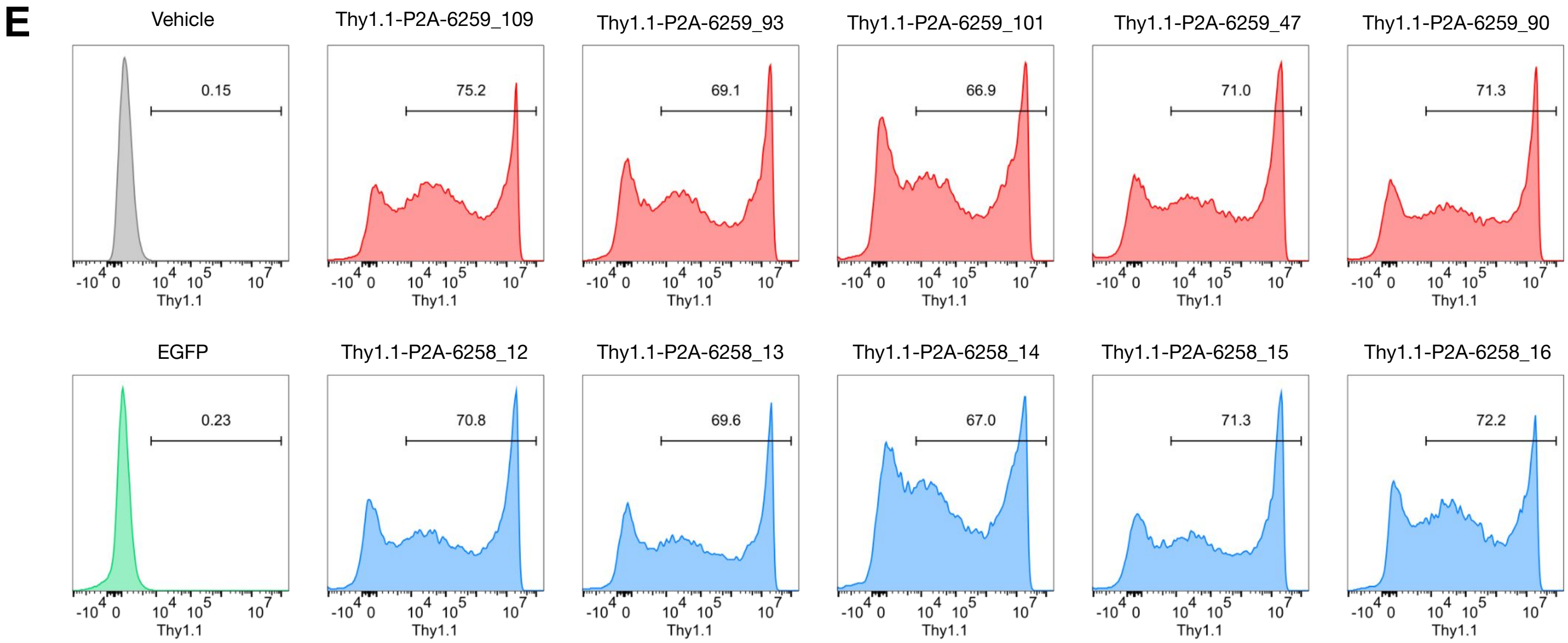

Table S1

bioRxiv preprint doi: https://doi.org/10.64898/2026.10.24.761497; this version posted October 27, 2026. The copyright holder for this preprint (which was not certified by peer review) is the author/funder, who has granted bioRxiv a license to display the preprint in perpetuity. It is made available under a CC-BY 4.0 International license.

Design variants	Peptide number with EL-Rank <0.5% for 9-11 mers	sequence
6258_12	0	MSEQHIKDFLEQFYTALDAGDAETAAGLFEDGVRIHLWTGVEFTTQEQFRSWFESLFKTSENAHREILSLKVEGDKVEVEVRLEADFNGKKEVVNLKHEWVFEGDKVVEVRVRIFPE
6258_13	0	MSEEAIRAFLDRFYAALDAGDADTAAGLFADGVEIQLWTGVTFTTQDQFREWFRKLFATSENASRRILSLEVDGNRVVVDVRLTADIKGTPVEVDLRHKWWFEGDRVVRVEVQIRPL
6258_14	0	MSEEHIRQFLEKFYSALDAGDADTAAGLFKDGVKITLWTGVEFTTQEEFRDWFRKLFALSEDAHREILSLRVDGNEVTVRVRLRARFKGRPFEVDLEHRWHFEGDEVVEVEVRIFPV
6258_15	0	MSEQHIRDFLEQFYAALDAGDATTAAGLFEDGVKIHLWTGVEFTTQAEFRDWFETLFAQSENAAREITSLEVRGDEVTVHVRLRADFRGQPHEVDLEHRWRFEGDRVVEVEVRIFPL
6258_16	0	MSEEAIRDFLDRFYTALDAGDAETAAGLFEDGVRIHLWTGVEFTTRAEFREWFERLFAQSEAARRRILSLEVDGDTVHVDVELRALIQGRPVRVRLRHTWRFEGDRVVEVRVEIFPV
6259_47	25	MTKAEIQAFLNKFYTALDNGNSVEASSLFENGVTIELWNGVIFTSPQQFKDWFDKLFSISSNAKRTIKDLKVDGNKVSVVVHLTAIVNGQEVNVDLLHEWTFENGKVKSVKVTILPK
6259_90	21	MTKAEIVAFLNTFYTALDNKDSVAAASLFENGVKIKLWNGVEFSSPQEFRDWFDELFKISSNAKRTIVSLEVNGNVVVVDVLLEATLDGKKISVRLRHVWTFENGKVAAVNVDIIPL
6259_93	32	MSVQQIRDFLTKFYTALDNGDSKGAAALFENGVTITLWNGVTFRNPKAFENWFDELFKVSADANREILSLSVNGNTVTVDVRLRAIVNGEALNVVLRHVWTFEDGKVKAVKVTIIEA
6259_101	29	MTQAEIKSFLNKFYTALDNKNSVAASSLFENGVKIKLWTGVEFNSPEEFEAWFNELFKISSNAKRTIVSLSVNNNVVIVDVHLTAVVNGENLDVMLRHIWTFENGKVKEVVVEIIPK
6259_109	15	MSLEQIREFLARFYAALDAGDADGAAALFANGVEIELWDGVRFTNPQAFRDWFRELFRRSRDARREIIALKVDGDTVTVVVRLRAIVNGEELDVLLRHVWTFRDGRVAAVRVEIEPL
